# Success and opportunities: maternal mortality and health services in Sierra Leone

**DOI:** 10.3389/fgwh.2026.1613691

**Published:** 2026-04-10

**Authors:** Kassim Kamara, Bridget Magoba, Tom Sesay, Zainab Bah, Musu Cole, Awol Yemane, Francis Moses, Gebrekrstos Negash Gebru

**Affiliations:** 1 African Field Epidemiology Network, Freetown, Sierra Leone; 2 Ministry of Health, Freetown, Sierra Leone; 3 Aberdeen Women’s Center, Freetown, Sierra Leone; 4 Sierra Leone Field Epidemiology Training Program, Freetown, Sierra Leone

**Keywords:** antenatal care (ANC), disparities (health, health service, maternal morality, Sierra Leone

## Abstract

**Introduction:**

Sierra Leone has made significant improvements in maternal mortality reduction; however, disparities in maternal healthcare delivery services still exist. This analysis examined the characteristics and disparities in maternal health service indicators and mortality data. The findings guided evidence-based public health interventions and informed the monitoring of their effectiveness.

**Methods:**

We conducted a secondary analysis of maternal deaths and health services data reported in the National Maternal Deaths Surveillance and Response (MDSR) system and the District Health Information System (DHIS2) and supplemented it with line lists using Microsoft Excel 2016.from 2019 to 2024. We excluded uninvestigated deaths or deaths with missing key variables. Using QGIS version 3.12.2, and Microsoft Excel 2016, descriptive and spatial analyses were conducted to examine patterns and disparities in maternal health indicators, with findings presented in tables, graphs, and maps.

**Results:**

Overall, Maternal Mortality Rate (MMR) for the six years was 216 deaths per 100,000 live births, whereas the MMR from January through November 2024 was 183.8 per 100,000 live births (95% CI: 166–201.6). The MMR declined over the period under review, from 241 per 100,000 live births in 2019 to 184 per 100,000 in 2024. Skilled Birth Attendance (SBA) remained high, averaging 95% throughout the six years, while 36% women (*n* = 119) attended four or more antenatal care visits. Approximately 40% (*n* = 769) of the maternal deathsresulted from postpartum hemorrhage.

**Conclusion:**

In Sierra Leone, maternal mortality declined over the study period; however, disparities in service utilization persist. While SBA remains high, coverage of at least 4 ANC visits was suboptimal. Postpartum hemorrhage (PPH) was the leading cause of maternal death. Targeted efforts to improve access to quality antenatal and delivery services in high-burden districts, are essential to accelerating reductions in maternal mortality.

## Background

Maternal death is the death of a woman while pregnant or within 42 days of termination of pregnancy from any cause related to or aggravated by the pregnancy or its management, but not from accidental or incidental causes (1).

Globally, according to the World Health Organization (WHO), approximately 830 women die each day from complications related to pregnancy or childbirth. Ninety-nine percent of all maternal deaths occur in developing countries, particularly among women in rural areas and low-income communities. Compared with that in developed nations, the high number of maternal deaths in low- and middle-income countries reflects disparities in access to quality healthcare services. In low-income countries, there are approximately 430 maternal deaths per 100,000 live births, whereas high-income countries experience only 12 deaths per 100,000 live births (2).

The WHO recommends that every woman have at least eight antenatal care (ANCs) contacts during pregnancy (3). Globally, 88% of women receive at least one ANC visit, but only 69% receive at least 4 ANC visits (4). However, ANC utilization is lower in sub-Saharan Africa (SSA), with 49%, 55.5%, and 58.5% of studies reporting at least 4 visits.

Approximately 81% of births worldwide are attended by skilled professionals, whereas only 60% in sub-Saharan Africa are???? (attended by skilled professionals??) (5).

Globally, the leading causes of maternal mortality include hemorrhage, infections, hypertensive disorders, abortion complications, and complications during delivery (6). In SSA, the major direct causes of maternal death include hemorrhage (34%), infection (10%), hypertensive disorders (9%), and obstructed labor (4%) (7).

In a study conducted in Sierra Leone, the leading causes of maternal death were obstetric hemorrhage (39.4%), hypertensive disorders (15.8%), and pregnancy-related complications (10.1%) (8). According to the 2019 Sierra Leone Demographic and Health Survey (DHS) report, Sierra Leone has one of the highest maternal mortality ratios (MMRs) in the world, with 717 deaths per 100,000 live births (9). However, recent United Nations and World Bank estimates indicated a considerable decrease in MMR to 443 deaths per 100,000 live births compared with the 2019 DHS MMR report (10).

Despite the substantial reduction in the country's maternal mortality rate (MMR), challenges persist, similar to those faced by many low-income countries. These challenges include disparities in maternal healthcare services and poor data quality, which hinder the formulation of effective policies and evidence-based actions. Furthermore, thorough analyses of maternal health service indicators using the available data are limited.

Analyzing maternal health service indicators is essential for informing public health actions. The Sierra Leone MoH, through the Directorate of Reproductive and Child Health, conducted this analysis with funding and technical support from Vital Strategies and the African Field Epidemiology Network (AFENET). This analysis examined the characteristics and disparities in maternal health service indicators and mortality data. The findings guide evidence-based public health interventions and can serve as a baseline for monitoring their effectiveness.

## Materials and methods

### Study design and period

We conducted a descriptive study using secondary maternal mortality data reported for six years. This study was conducted from November 2024 to April 2025.

### Study setting

Sierra Leone is a country in West Africa with a population of 7,874,335. Administratively, the country is divided into four regions, sixteen districts, and 190 chiefdoms (11). It comprises over 1,585 public and private health facilities organized into primary, secondary, and tertiary levels. All health facilities in the country provide maternal health services.

### Data collection

For this analysis, we obtained live birth data from the National Health Management Information System (NHMIS) running on the District Health Information System (DHIS2) platform from 2019 through 2024, using Microsoft Excel 2016. Information on maternal deaths from 2019 to 2023 was obtained from the national maternal death line list in MS Excel. In contrast, data for 2024 were extracted from the Electronic Case-Based Disease Surveillance (eCBDS) system running on the DHIS2 platform in Microsoft Excel format. The causes of maternal death are determined and recorded during maternal death reviews by a committee of maternal healthcare providers, including medical doctors, midwives, surveillance officers, and other clinicians, using the International Classification of Diseases, Tenth Revision (ICD-10) for the immediate cause of death (the event that directly led to death), the underlying cause of death, summary contributory condition 1, summary contributory condition 2, and summary contributory condition 3. We extracted data on demographics, parity, antenatal care (ANC) visits, cause of death, and mode of delivery, among other variables.

### Data management and analysis

We calculated the maternal mortality ratio (MMR) by dividing the number of maternal deaths by the number of live births, then multiplying by 100,000. We excluded deaths that were not investigated or those with key missing variables, such as ANC coverage, age, parity, and cause of death, from the analysis of the characteristics of maternal deaths. The key variables analyzed included socio-demographic variables (age, level of education, place of death, and time of death), number of ANC visits, parity, mode of delivery, and cause of death. Discritpitive and spatial analysis were analyzed using QGIS version 3.12.2, MS Excel 2016, and SPSS. We conducted the descriptive and spatial analysis using frequencies and proportions. The MMR was computed using the number of maternal deaths as the numerator, the number of live births as the denominator, and 100,000 as a constant. We analyzed categorical variables using frequencies and percentages. For each proportion, we calculated 95% confidence intervals to estimate the precision of the observed estimates. The analysis was descriptive and conducted at the univariate level.. The data were summarized and presented in tables, charts, graphs, and maps.

#### Ethical consideration

This study examined anonymized secondary data obtained from routine public health surveillance systems and maternal surveillance and response systems to ensure compliance with ethical standards. The MoH leadership approved the analysis and use of these data, and individual patient consent was not needed. The study followed ethical guidelines for sharingpublic health surveillance data, in line with World Health Organization (WHO) recommendations.

## Results

Overall, the MMR for the six years (2019–2024) was 216 deaths per 100,000 live births. From January through November 2024, there were 220,311 live births and 405 maternal deaths, resulting in an MMR of 183.8 per 100,000 live births (95% CI: 166–201.6). Of the 405 maternal deaths from January through November 2024, only 81% (329) had complete records after incomplete records were excluded from the quantitative analysis but included in the gap analysis.

Table 1 shows the characteristics of maternal deaths investigated from January to November 2024. The average age of maternal death was 28 years (range: 16–42). The majority of maternal deaths occurred among women aged 20–34 years, 68% (223), with secondary education or lower, 60% (196). The majority of the deaths, 77% (254), occurred in health facilities, whereas 62% (203) occurred after delivery.

**Table 1:** Demographic and obstetric characteristics of maternal deaths investigated, Sierra Leone, January to November 2024 per 100,000 live births, *N* = 329.

Characteristic	Number	Percent	95% CI
Age group (years)			
≤19	44	13	9.6–17.4
20 to 34	223	68	62.7–72.8
≥35	62	19	15.1–24.3
Level of education			
Secondary school and lower	196	60	54.4–65.2
Tertiary	18	6	3.6–9.4
Unknown	115	36	31.0–41.1
Place of death			
Health facility	254	77	72.5–81.3
Community	27	8	5.4–11.5
Transit	18	6	3.6–9.4
Unknown	30	9	6.4–12.7
Time of death			
Before delivery	61	18	14.1–22.9
During delivery	29	9	6.4–12.7
After delivery	203	62	56.4–67.5
Unknown	36	11	8.0–15.1

Table 2 describes the obstetric characteristics of maternal deaths investigated from January to November 2024, with only 36% (119) having attended four or more ANC visits. Compared with their counterparts, multiparous women accounted for more deaths, with 37% (121).

**Table 2:** Obstetric characteristics of maternal deaths investigated, Sierra Leone, January to November 2024 per 100,000 live births, *N* = 329.

Characteristic	Number	Percent	95%CI
Number of ANC visits			
Zero (0)	41	12	8.89–16.03
1 to 3	115	35	29.80–40.11
≤4	119	36	30.98–41.36
Unknown	54	16	12.41–20.42
Parity			
Nulliparous	44	13	9.70–17.05
Primiparous	60	18	14.06–22.41
Multiparous	121	37	31.57–41.99
Grand multiparous	73	22	17.70–26.68
Unknown	31	10	6.27–12.58

### Selected birth characteristics

**Figure 1:**
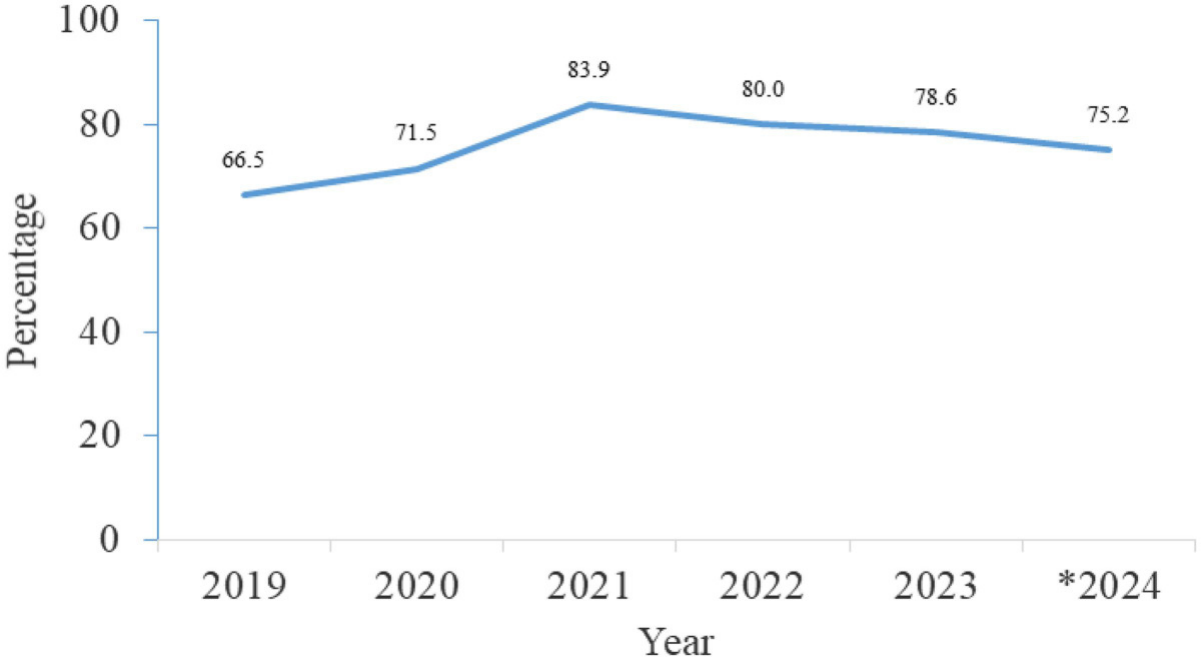
Proportion of women who made at least 4 ANC visits per year, Sierra Leone, from January 2019 to November 2024. *2024: This year runs from January to November 2024.

Figure 1 illustrates the trend in the annual proportion of women who made at least 4 ANC visits at the population level in Sierra Leone. Overall, the percentage of 4th ANC visits improved over the six years, averaging 76%. The highest coverage was recorded in 2021 at 84%, whereas the lowest was recorded in 2019 at 67%. These data reflect the utilization of ANC services and the relative success of the healthcare system in retaining pregnant women for multiple visits.

**Figure 2:**
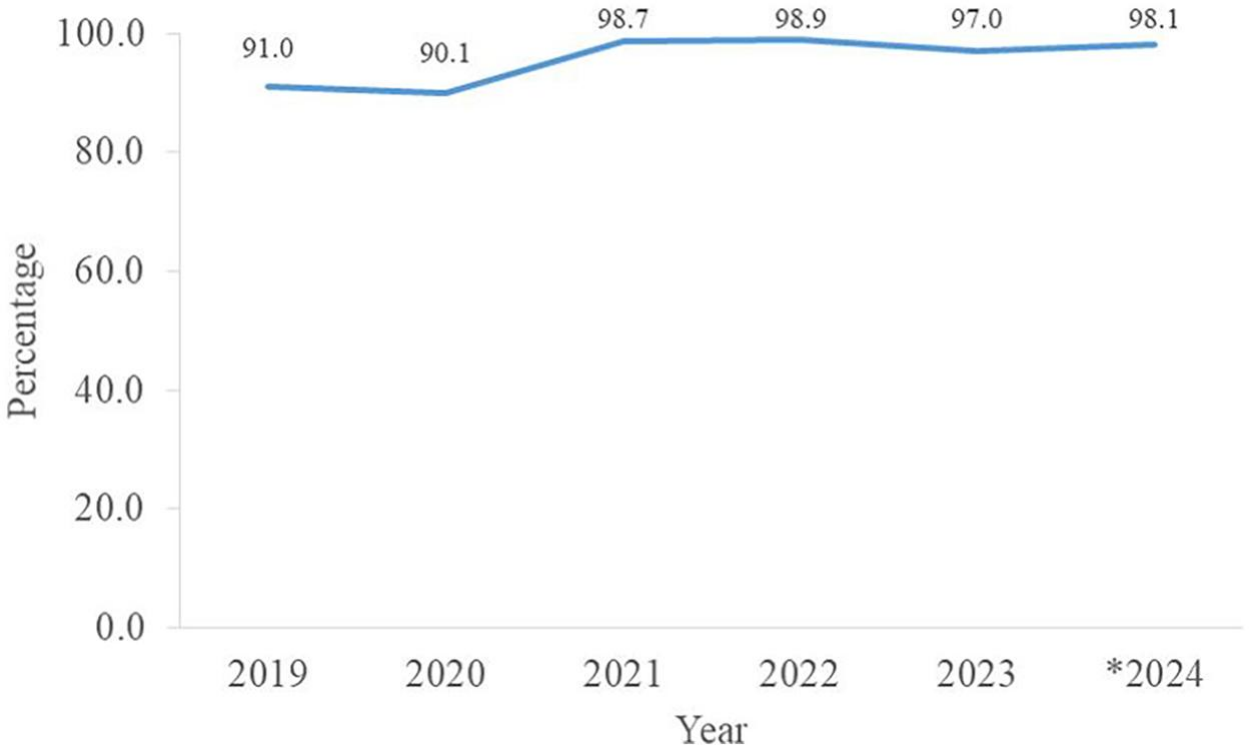
Proportion of deliveries attended by skilled birth attendants by year, Sierra Leone, January 2019 to November 2024, *N* = 1,351,822. *2024: This year runs from January to November 2024.

Figure 2 illustrates the annual proportion of deliveries attended by skilled birth attendants, indicating access to qualified maternal care during childbirth. There was a gradual increase in the proportion of deliveries attended by skilled birth attendants, from 89% in 2019 to 99% in 2024.

### Maternal deaths

Table 3 shows the prevalence of eclampsia among live births, the number of maternal deaths, and the maternal mortality ratio by year for the study period, with a gradual decrease in the MMR from 2019 to 2024.

**Table 3:** Live births, maternal deaths, and maternal mortality ratios by year, Sierra Leone, 2019 to 2024.

Year	Live births	Maternal deaths	MMR	95%CI
2019	235,437	579	246	225.9–266.0
2020	231,343	542	234	214.6–254.0
2021	218,345	506	232	211.6–252.0
2022	217,432	462	212	193.1–231.9
2023	241,017	457	190	172.3–207.0
*2024	220,311	405	184	165.9–201.7

The symbol * indicates that the 2024 data covered January to November.

**Figure 3:**
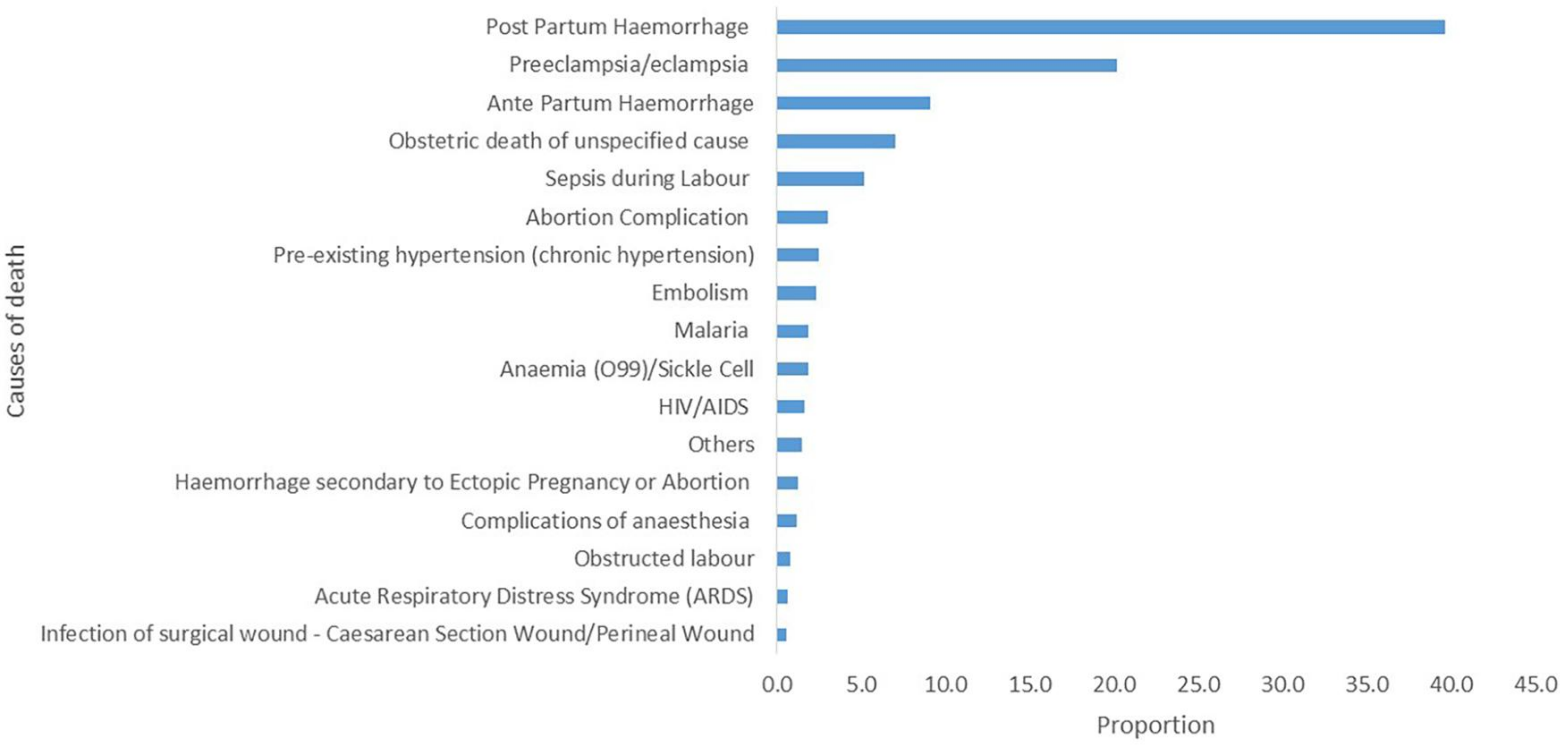
Leading causes of maternal deaths, Sierra Leone, January 2019 to November 2024, *N* = 2,005.

Among the most frequently reported causes of maternal death (Figure 3), postpartum hemorrhage accounted for 40% (796), followed by pre-eclampsia/eclampsia, with 20% (405) accounting for the top two leading causes of death. In contrast, acute respiratory distress syndrome (0.6%, 12) and infection of the surgical wound (cesarean section wound/perineal wound) (0.5%, 11) were the least common causes of maternal death.

**Figure 4:**
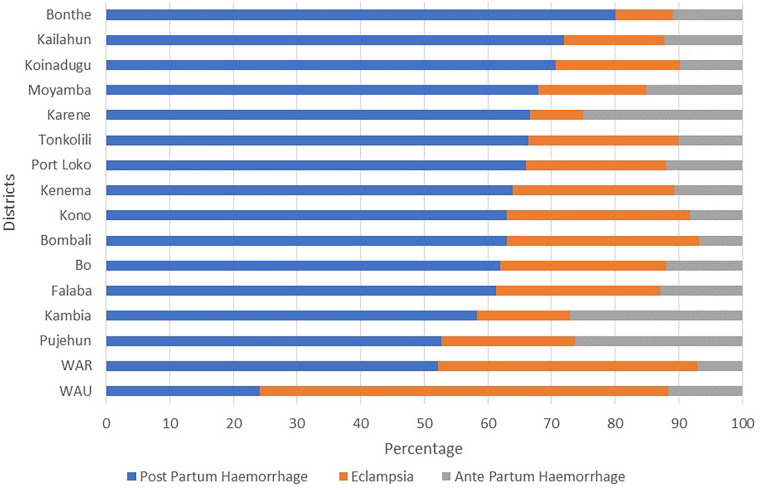
Proportion of the three most reported causes of maternal mortality by district, Sierra Leone, 2019–2024.

Figure 4 compares the three most reported causes of maternal death across districts. In Bonthe district, 80% (44) of maternal deaths were due to postpartum hemorrhage. The same pattern was also observed in all the other districts, except in the western urban area, where pre-eclampsia/eclampsia (64%, 138) caused more maternal deaths than did postpartum hemorrhage and antepartum hemorrhage.

**Figure 5:**
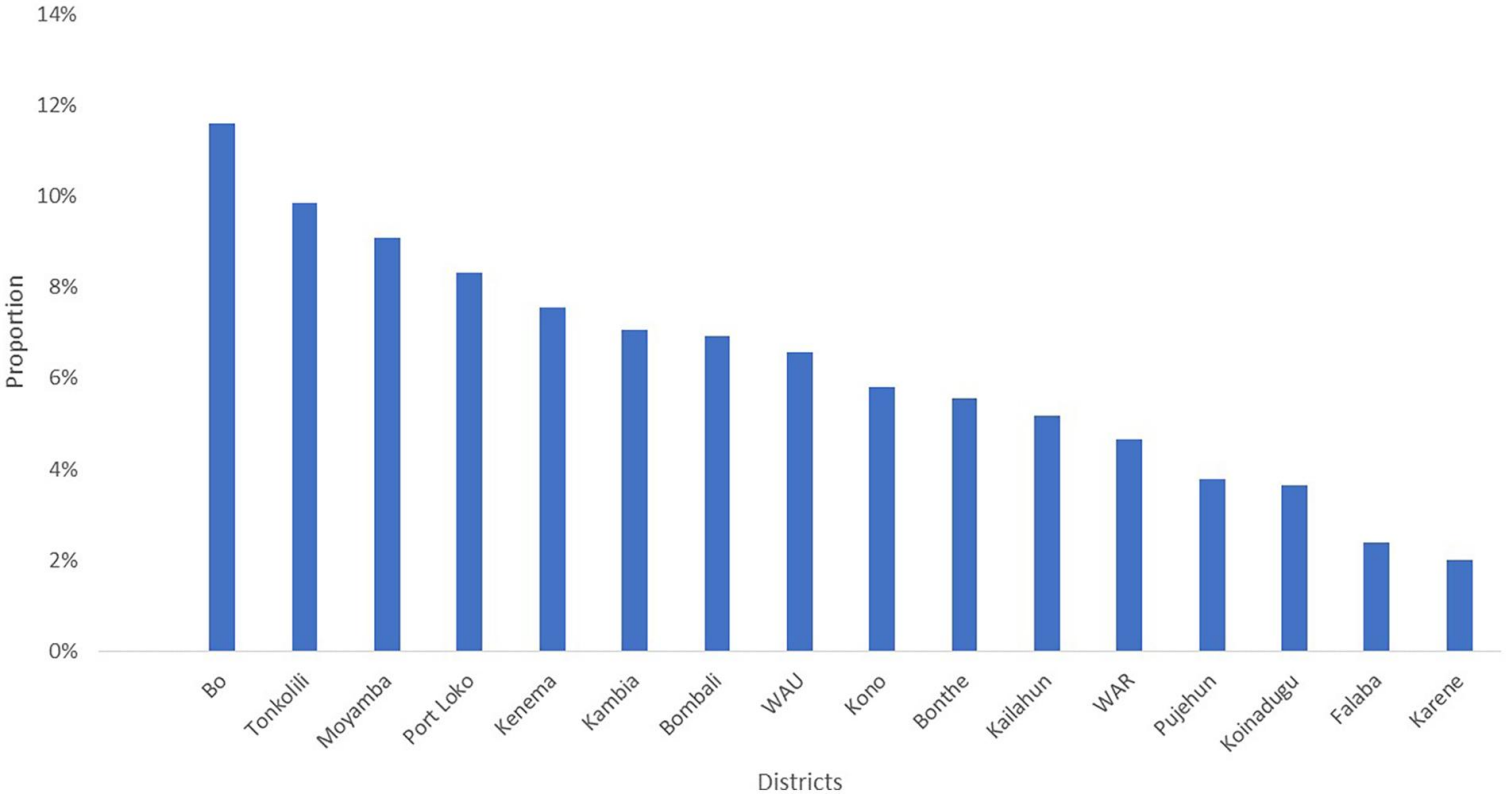
Leading cause (hemorrhage) proportion of maternal death by district, Sierra Leone, January 2019 to November 2024, *N* = 793.

Figure 5 shows the distribution of hemorrhage as a leading cause of maternal death by district from January to November 2024. Bo District, followed by Tonkolili District, recorded higher proportions of overall hemorrhage-related deaths, with 12% (92) and 10% (78), respectively. On the other hand, Falaba district (2%, 19) and Karene district (2%, 16) recorded the lowest proportions of hemorrhage as a cause of death compared with the other districts. Regarding the regional pattern, the highest proportion, 30.8% (228), was from the Southern region (Bo, Pujehun, Bonthe, and Moyamba), followed by the Northern region with 20.3% (150).

**Figure 6:**
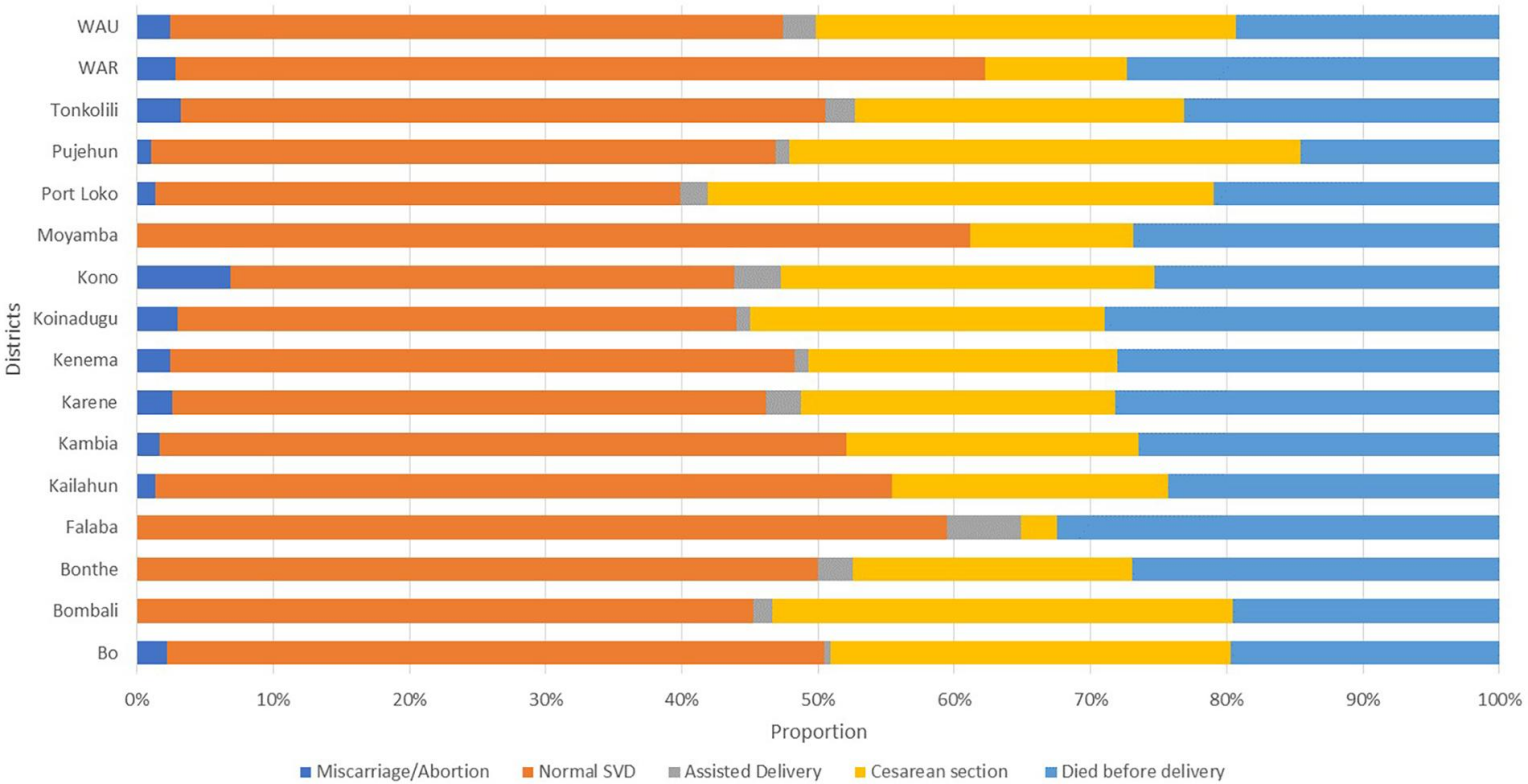
Proportion of maternal deaths by mode of delivery by district, Sierra Leone, January 2019 to November 2024, *N* = 2,179.

Figure 6 illustrates the proportion of maternal deaths by mode of delivery by district. The graph shows that normal spontaneous vaginal delivery (SVD) accounted for the majority of maternal deaths in all the districts, with higher proportions reported in Moyamba district, 61% (82); Falaba, 59% (22%); and Western Area Rural, 59% (63). Among the total number of maternal deaths reported in the districts, the number of cesarean section-related deaths per district was much greater in some districts, such as Pujehun, which accounted for 38% (36), and the Port Loko district, which accounted for 37% (55).

**Figure 7:**
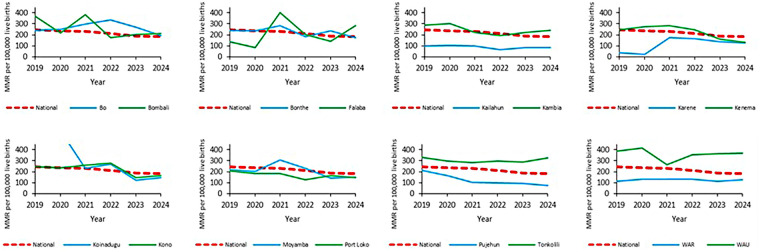
Maternal mortality ratio by district and year, Sierra Leone, January 2019 to November 2024.

Figure 7 presents the trend in MMR by district relative to the national ratio. Overall, the national MMR declined gradually from 2019 to 2024, paralleling improvements in maternal health service indicators. However, the MMR varies significantly across districts. Districts such as Karene, Western Area Rural, and Kailahun had low MMR values in 2019 and 2020. In contrast, other districts, such as Kenema and Kambia, showed relatively steady or fluctuating trends during the analysis period. The Tonkolili and Western Area urban districts reported consistently high MMRs throughout the six years, which is indicative of continuing challenges in addressing maternal mortality in these areas. The Bo and Bombali districts, on the other hand, recorded relatively stable and lower MMR trends.

**Figure 8:**
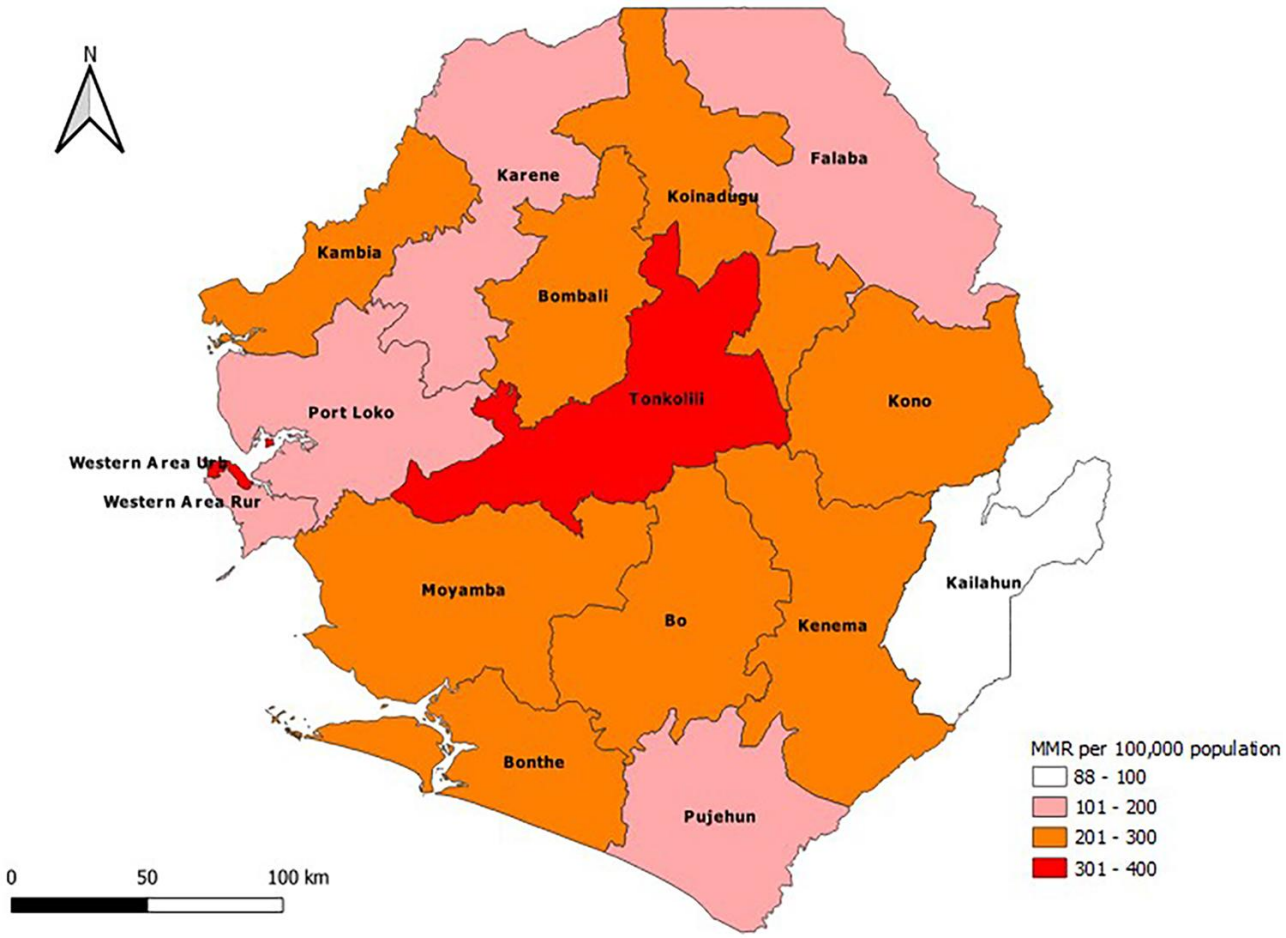
Maternal mortality ratio by district by year, January 2019 to November 2024.

As shown in Figure 8, the Western Area Urban district, followed by the Tonkolili district, recorded the highest MMRs, with 358 (329.9–389.4) and 304 (267.0–341.0) deaths per 100,000 live births, respectively. On the other hand, the Kailahun and Pujehun districts recorded lower MMRs, ranging from 88 (71.4–104.9) to 123 (101.2–144.9) deaths per 100,000 live births.

**Figure 9:**
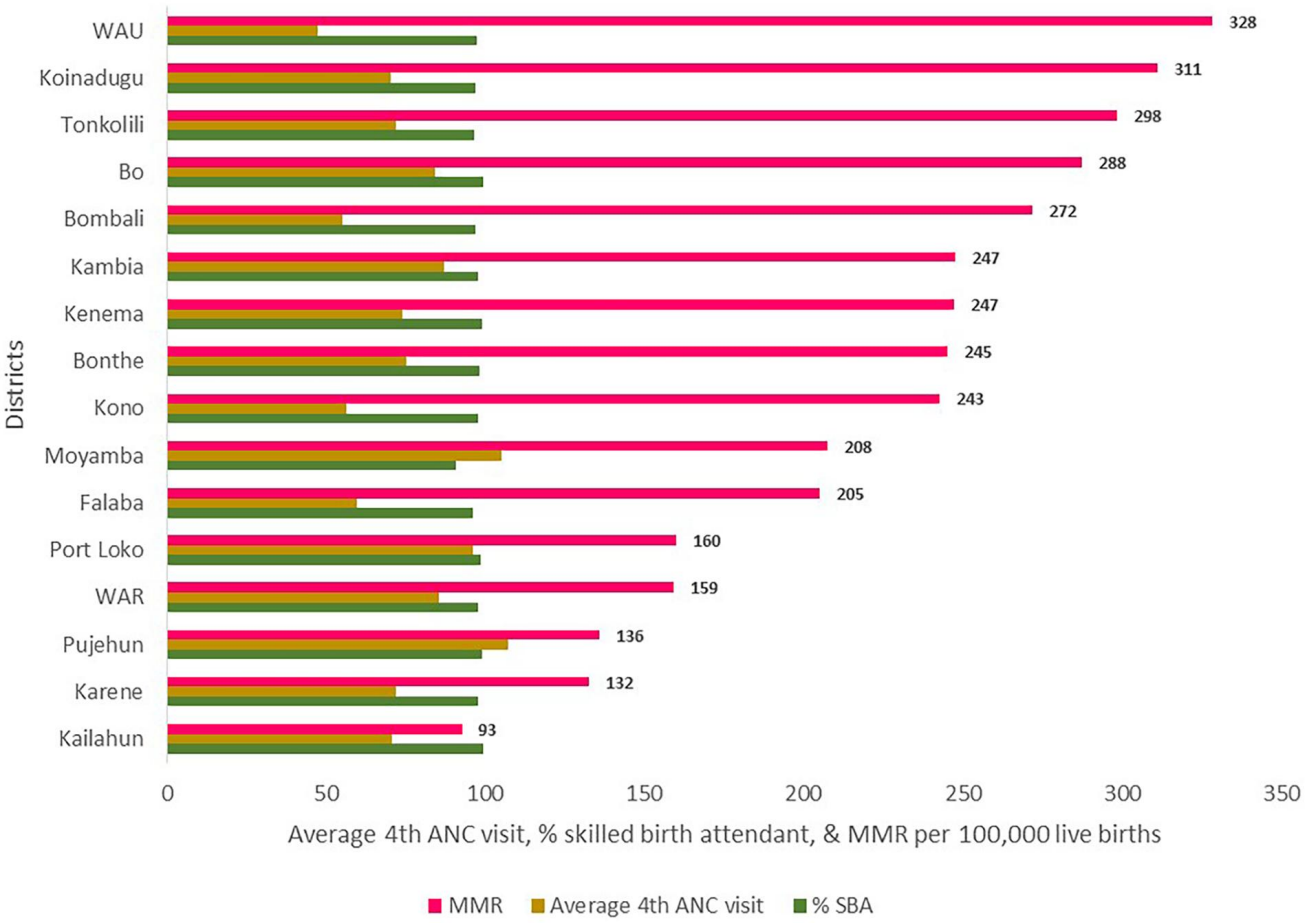
Average percent receiving four or more ANC visits, SBAs, and MMRs by district, Sierra Leone, 2019–2024.

Figure 9 shows an inverse relationship between the average proportion of women receiving four or more ANC visits, skilled birth attendance (SBA), and the maternal mortality rate (MMR). Districts with lower ANC coverage recorded higher MMRs. However, the SBA rate is high across the districts, with Moyamba the only district below 95%. For example, the western urban area recorded the highest MMR of 328 per 100,000 live births and one of the lowest average coverages for four or more ANC visits at 48%. In contrast, the Karene and Kailahun districts presented relatively high ANC coverage rates of 72% and 71%, respectively, and lower MMRs of 132 and 93 per 100,000 live births, respectively. Moyamba, however, stands out for having an average of four or more ANC visits of 105% and a high MMR of 108 per 100,000 live births, suggesting that other factors contribute to maternal mortality. There are four districts where the proportion of pregnant women with four or more ANC visits is 60% or lower.

## Discussion

This analysis reported an MMR of 183.8 per 100,000 live births from January 2024 through November 2024. Furthermore, it describes the characteristics and disparities in maternal health service indicators and mortality trends in Sierra Leone, revealing that ANC visit coverage at the population level started increasing since 2019 and approaching 80%. This represents a significant success, since attending 4 or more ANC visits is associated with reduced maternal mortality (12, 13). However, this ANC coverage reflects the population-level pattern, and may therefore not directly correlate with a reduction in maternal mortality in the current data, since some pregnant women may not receive the required services during their visits to health facilities. Furthermore, this study could not determine the ANC coverage among maternal deaths due to the unavailability of the pregnant women population during the study period. The efforts of the MoH to provide pregnant women with access to ANC and retain them in care for multiple visits are important for improving pregnancy outcomes. However, the district-level analysis shows that four districts are still far from achieving this goal, with only 48%–60% of women receiving 4 or more ANC visits. This could have consequences such as late detection of pregnancy danger signs, low coverage on pregnancy-related services received, which could result in increased maternal mortality in those districts (14, 15). Efforts to address these barriers to antenatal care in these areas should be prioritized.

This study also reported that more than two-thirds of maternal deaths were among those aged 20- 34 years. Similarly, studies on maternal mortality conducted in Tanzania, South Africa, Ghana, and Nigeria reported a high proportion of maternal mortality among those aged 20–34 years (16–19). Possible reasons for this consistent finding across other studies include the fact that this age category is when most women give birth in Africa, suggesting that studies should be conducted to examine other factors influencing this pattern. The report from this study that the majority of maternal deaths occurred in health facilities could be attributed to the increase in health facility delivery, considering the efforts put in by the MoH to increase health facility delivery (20). Furthermore, since some community maternal deaths are not usually reported, the extent of this has not been established. In contrast, maternal deaths occurring in health facilities or during transit are mostly reported, which could account for the disparity by place of occurrence. However, while the MoH continues to promote health facility delivery, it is imperative to also improve the quality of service delivery on maternal health, such as the availability of adequate skilled personnel and medical supplies, to reduce health facility-related deaths.

The increasing percentage of deliveries attended by skilled birth attendants (SBAs) during the review period, now approaching 100%, demonstrates the ongoing and successful efforts of the MoH to train and deploy SBAs across health facilities nationwide (21). However, there are disparities in this indicator across districts, with urban areas performing better than rural areas. This may be attributed to several factors, including the concentration of health facilities, personnel, and greater access to behavior change messages in urban regions (22).

Like other studies, this analysis revealed that postpartum hemorrhage was a leading cause of maternal death (23). The high proportion of maternal deaths associated with hemorrhage may suggest an opportunity to strengthen emergency obstetric care. This finding points to a potential need for targeted interventions, such as providing antenatal and postnatal care services in health facilities (24). Other investigators have reported findings on the role of postpartum hemorrhage as the leading cause of maternal death in the country (25, 26).

Although PPH has been reported as the leading cause of maternal death in almost all districts, pre-eclampsia/eclampsia is the leading cause of death in Western Area Urban. This finding could be attributed to the greater availability of health facilities equipped with specialized clinicians and blood bank services, among other services, in Western Area Urban to manage PPH-related cases than in other districts (27), a study in Africa reported that aspects of pregnancy and delivery, PPH management, and organizational characteristics of hospitals are determinants of hospital-based PPH maternal death (28). On the other hand, while the high proportion of maternal deaths due to pre-eclampsia/eclampsia in Western Area Urban might be influenced by lifestyle factors, as suggested by other studies (29–31), this analysis did not directly measure these variables. Therefore, further investigation is needed to determine if urban lifestyle patterns significantly contribute to hypertensive disorders in this population.

The high proportion of maternal deaths among women who had normal SVD could be attributed to the high number of SVDs compared with other modes of delivery. Furthermore, districts with high proportions of maternal deaths from cesarean could experience surgical complications or delays in seeking care (32). However, there could be underlying causes for the high proportion of maternal deaths that had SVD, especially in the Moyamba, Falaba, and Western Area Rural districts, and the variation in maternal deaths among women delivered via via cesarean section need further exploration to understand the underlying causes of deaths related to SVD and other modes of delivery to guide evidence-based interventions.

Even though Sierra Leone still has a high MMR, the gradual decline in the national MMR underscores the commitment and efforts of the MoH and its partners through the implementation of evidence-based actions to reduce maternal mortality. Despite the overall gradual decrease in the MMR, districts such as Tonkolili and Western Area Urban should receive special attention since they recorded consistently high MMRs throughout the period under review. Furthermore, additional research to understand the factors driving variations between districts should be conducted, and appropriate measures should be taken to reduce the MMR, especially in the most affected districts.

These variations in MMR across districts may indicate disparities in access to healthcare services, the quality of services provided, or the effectiveness of district-specific maternal health activities. These disparities call for further studies to identify their associated factors and guide evidence-based interventions to address the specific challenges faced by high-burden districts. At the same time, efforts are maintained in districts with declining trends.

However, the variation in MMR across districts may also partly reflect differences in reporting completeness and in the performance of the surveillance system, since the analysis relied on routinely collected national surveillance data.

### Limitations

We could not estimate the MMR for certain variables, such as age category, education level, marital status, mode of delivery, and others, because we lacked case-based live birth data to serve as denominators. Notably, the results from this analysis may underrepresent the actual burden of maternal mortality due to the underreporting of maternal deaths from communities.

## Conclusion

This analysis revealed declining MMR, which could be attributed to improved postnatal care and skilled birth deliveries from 2019 to 2024. The Northern region reported a higher MMR, while the Southern region showed better performance at the 4th ANC visit than other regions. Several factors may account for the disparity among regions, including low ANC coverage and limited availability of blood bank services, suggesting the need for further research to obtain the necessary information for appropriate public health actions.

The MoH and its partners should continue improving access to ANC and SBAs, especially in highly affected districts. In addition, the MoH should increase access to quality healthcare services by allocating additional resources and improving service quality in these districts to address gaps in maternal healthcare and reduce disparities in maternal health services. Finally, the MoH should explore district-specific inequalities in maternal health outcomes, collaborate with research institutions to collect the necessary evidence to understand and monitor the factors most affecting maternal mortality in Sierra Leone, and develop evidence-based interventions to improve maternal health in the country. Qualitative research may explore the variance in maternal service inequalities across districts, and quantitative studies may estimate the actual burden of maternal deaths at the community level.

## Data Availability

The raw data supporting the conclusions of this article will be made available by the authors, without undue reservation.
